# Do women ⩾50 years of age need as much screening as women <50 years after they have had negative screening results?

**DOI:** 10.1038/sj.bjc.6604455

**Published:** 2008-07-01

**Authors:** P Armaroli, F Gallo, A Bellomi, S Ciatto, D Consonni, D Davi, P Giorgi-Rossi, A Iossa, E Mancini, C Naldoni, E Polla, G Ronco, M Serafini, V Vergini, L Zanier, M Zappa, N Segnan

**Affiliations:** 1 SCDO Epidemiologia dei Tumori, Centro Prevenzione Oncologica Regione Piemonte and Azienda Ospedaliero-Universitaria S. Giovanni Battista di Torino, V. San Francesco da Paola 31, Torino 10123, Italy; 2 SCDO Epidemiologia dei Tumori, Centro Prevenzione Oncologica Regione Piemonte and Azienda Ospedaliero-Universitaria S. Giovanni Battista di Torino, V. San Francesco da Paola 31, Torino 10123, Italy; 3 Servizio di Anatomia Patologica, Ospedale C Poma, Via Al Lago 7, Curtatone, Mantova 46010, Italy; 4 Unità di Diagnostica per Immagini, Istituto Scientifico Prevenzione Oncologica, Viale A. Volta 171, Firenze 50131, Italy; 5 Unità Operativa di Epidemiologia, Fondazione IRCCS Ospedale Maggiore Policlinico Mangiagalli e Regina Elena, Via San Barnaba 8, Milano 10122, Italy; 6 Dipartimento di Sanità Pubblica, Azienda Unità Sanitaria Locale di Ferrara, Via F. Beretta 7, Ferrara 44100, Italy; 7 Laziosanità Agenzia di Sanità Pubblica, Regione Lazio, Via Santa Costanza 53, 00198 Roma, Italy; 8 Unità Operativa Prevenzione Secondaria Screening-CRR, Istituto Scientifico Prevenzione Oncologica, Viale A. Volta 171, Firenze 50131, Italy; 9 Unità di Valutazione e Organizzazione Screening, Centro Prevenzione Oncologica Regione Piemonte and Azienda Sanitaria Locale TO1, V. San Francesco da Paola 31, Torino 10123, Italy; 10 Assessorato Politiche per la Salute, Regione Emilia-Romagna, Viale A. Moro 21, Bologna 40127, Italy; 11 Istituto di Anatomia Patologica, Ospedale Santa Chiara, Largo Medaglie d’Oro 1, Trento 38100, Italy; 12 SCDO Epidemiologia dei Tumori, Centro Prevenzione Oncologica Regione Piemonte and Azienda Ospedaliero-Universitaria S. Giovanni Battista di Torino, V. San Francesco da Paola 31, Torino 10123, Italy; 13 Centro di Prevenzione Oncologica, Azienda Unità Sanitaria Locale di Ravenna, Italy, Viale Randi 5, Ravenna 48100, Italy; 14 SCDO Epidemiologia del Tumori, Centro Prevenzione Oncologica Regione Piemonte and Azienda Ospedaliero-Universitaria S. Giovanni Battista di Torino, V. San Francesco da Paola 31, Torino 10123, Italy; 15 Agenzia Regionale di Sanità Friuli Venezia Giulia, Piazzale Santa Maria della Misericordia, Udine 33100, Italy; 16 Unità di Epidemiologia Clinico-descrittiva, Istituto Scientifico Prevenzione Oncologica, Viale A. Volta 171, Firenze 50131, Italy; 17 SCDO Epidemiologia dei Tumori, Centro prevenzione Oncologica Regione Piemonte and Azienda Ospedaliero-Universitaria S. Giovanni Battista di Torino, V. San Francesco da Paola 31, Torino 10123, Italy

**Keywords:** cervical intraepithelial neoplasia, false-positive cases, mass screening, middle aged, vaginal smears

## Abstract

To assess the adequacy of a routine screening to identify cervical intraepithelial neoplasia 2 or worse (CIN2+) in women over 50 years of age, a retrospective cohort was set in six Italian organised population-based screening programmes. In all, 287 330 women (1 714 550 person-years of observation, 1110 cases) screened at age 25–64, with at least two cytological screening tests, the first negative, were followed from their first negative smear until a biopsy proven CIN2+ lesion or their last negative smear. For women aged 25–49 and 50–64 years, crude and age-standardised detection rate (DR), cumulative risk (CR), adjusted hazard risk for number of previous negative screens, probability of false-positive CIN2+ after two or more smear tests were calculated. Detection rate is significantly lower over 50 years of age. Multivariable analysis shows a significant protective effect from four screening episodes (DR=0.70, 95% CI: 0.51–0.97); the effect of age ⩾50 is 0.29 (95% CI: 0.24–0.35). The CR of CIN2+ is at least eightfold higher in women <50 (CR=2.06, 95% CI: 1.88–2.23) after one previous negative test than in women ⩾50 years with four screens (CR=0.23, 95% CI: 0.00–0.46). Over 50 years of age, after four tests at least three false-positive cases are diagnosed for every true positive. Benefits arising from cytological screening is uncertain in well-screened older women.

European recommendations state that cervical cancer screening should start at the latest at 30 years and stop not before 60 years of age. Screening intervals should be between 3 and 5 years (Coleman *et al*, 1993).

International Agency for Research on Cancer recommendations for cervical cancer screening suggest 5-yearly screening over 50 years of age; 3-yearly screening is recommended at age 25–49 years in countries with the necessary resources (International Agency for Research on Cancer, 2005).

The overtreatment is intrinsic in cervical cancer screening: for preventing one invasive cancer, seven to eight preinvasive lesions should be treated (Ponten *et al*, 1995).

Considering invasive cervical cancer, several studies (International Agency for Research on Cancer, 1986; Gustafsson *et al*, 1995; Cruickshank *et al*, 1997; Coldman *et al*, 2005; Ronco *et al*, 2005) show a trend of decreasing risk with increasing number of smear tests.

Detection rates (DRs) for cervical intraepithelial neoplasia (CIN) fall with increasing age (Gustafsson *et al*, 1995; Gram *et al*, 1998) and number of previous smears (Gram *et al*, 1998).

The low risk level in well-screened women after 50 years of age might not justify the costs and harms associated with screening, such as overdiagnosis, anxiety, unnecessary treatment and reduction in quality of life associated with false-positive results (International Agency for Research on Cancer, 2005). On the basis of age and screening history, a subset of women could be identified, for whom the risk of preinvasive and invasive lesions is negligible and screening might be safely stopped before the currently recommended age limit (Sherlaw-Johnson *et al*, 1999) or different screening intervals might be adopted at different ages (International Agency for Research on Cancer, 2005).

The balance between the risk of being diagnosed with a preinvasive or invasive lesion and that of getting a false-positive cytohistological report must be discussed considering ethic and practical implications.

Objective of the present study is to estimate the DR and the cumulative risk (CR) of CIN2 or more severe lesions (CIN2+) and to compare it with the probability of false-positive episodes in women <50 or ⩾50 years of age, with at least two cytological screening tests, the first negative, as observed in six Italian organised population-based screening programmes.

## Materials and methods

### Study design

The study is based on a retrospective cohort of women screened at age 25–64 years, who had at least two cytological screening tests on 31 December 2000, the first being reported as negative. Women were recruited from six Italian organised population-based screening programmes (Ferrara, Florence, Mantova, Ravenna, Turin and Viterbo): women aged 25–64 years are invited every 3 years by mail; monitoring system and quality assurance have been established according to the European guidelines.

Women were followed from the date of their first negative smear test available in the archives until a biopsy proven CIN2+ lesion was diagnosed or until their last negative smear result (performed within 31 December 2000). We considered as a negative smear test all the smears either with a negative cytology or with a positive cytology followed by a negative biopsy proven CIN2+ diagnosis performed within 300 days from the smear test, as women are readmitted to routine screening. Follow-up continued for women with a less severe than CIN2+ histological diagnosis. The histological diagnosis was issued within 30 September 2001 following a smear test performed within 31 December 2000.

As different cytological classifications were used in screening centres, data were standardised according to a common cytological and histological classification (Bethesda NCI Workshop, 1993).

Negative smear tests performed within 300 days of the oldest available test for clinical or technical reasons were assumed as a unique screening episode, as recommended in screening protocols of the programmes that supplied data. To count the previous negative screening episodes, we individuated an index test for each woman. In a screening setting, the index test activates further assessments to ascertain the occurrence of the studied lesion; so we did not count it among the negative ones and we used it to define the time of exit from the cohort. For negative women, the last available negative smear was considered as the index test. However, for the cases, four different situations occurred: (1) the index test was the most recent smear performed within 300 days since the CIN2+ histological report, (2) when two or more tests were performed within 300 days of the positive histological result, the one with the worst diagnosis was chosen, (3) if the last smear test was performed at more than 300 days from the histological diagnosis, this test was identified as the index if positive, (4) again, if the last smear test was performed at more than 300 days before the histological diagnosis but was negative, the date of the histological diagnosis was considered as the date of the index test.

### Statistical analysis

Persons-years were computed from the date of the oldest negative smear test available in the screening history. For negative women, the index test was assumed as the end point of the observation period whereas for cases, the end point was the midpoint between the index test and the last available negative smear test, as we assumed that the lesion occurred on average in the midpoint of the interval between two tests.

Person-years were calculated according to time-dependent variables: the number of previous negative screening episodes (five categories: 1, 2, 3, 4 and 5+) and 5-year age groups.

Crude and age-standardised (direct method) CIN2+ DR was calculated as cases on person-years, CR, applying the exponential formula, and their confidence intervals at 95% (95% CI) were calculated for women of 25–49 and 50–64 years of age. It must be noted that the age intervals used to calculate the CR have different lengths. A Score test for trend of DRs by age class was computed (StataCorp, 2005).

For women with two or more smear tests, we estimated the joint probability of a false-positive CIN2+ diagnosis, when both cytology and the histology are false positive. We used the formula: 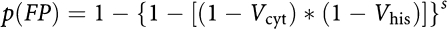 where *V*_cyt_ and *V*_his_ are the specificities of cytological and histological diagnosis and *s* is the number of screening episodes. We assumed the following combinations of cytological and histological specificities, respectively: 0.97–0.94, 0.96–0.95, 0.95–0.96 and 0.94–0.97 to calculate the number of false positives (FP). We estimated the probability of true-positive results (TP) as the probability of positive results (the CR per 10 000) minus the number of FP. The specificity values used are more favourable than the range of cytological and histological specificity shown in literature for HSIL/CIN2+ (Nanda *et al*, 2000; Stoler and Schiffman, 2001), and they represent the minimum values that combined together allow to estimate the TP number. We estimated the ratio between TP and FP, and the ratio between CR and FP.

The effect of multiple factors on the risk of CIN2+ was modelled using Cox regression for left-truncated and right-censored data. We have considered five categories of the number of previous negative screens and age <50 or ⩾50 years as time-dependent variables, whereas the interval between the index test and the last negative smear has been considered as a fixed variable. Calendar time was used as time axis. We calculated *P*-values for trend for the number of previous negative screens as an ordinal variable. *P*-values less then 0.05 were considered statistically significant.

Data were analysed through SAS (SAS Institute Inc., USA, 1999–2001) and STATA (StataCorp, 2005) softwares.

## Results

Screening data were available from the six programmes. Screening episodes, as defined in the Material and methods section, were 569 713. The study included 287 330 women for a total of 1 714 550 person-years of observation and 1110 CIN2+ cases.

Distribution of the study population and DR (95% CI) by screening programmes is described in Table 1.

**Figure 1:**
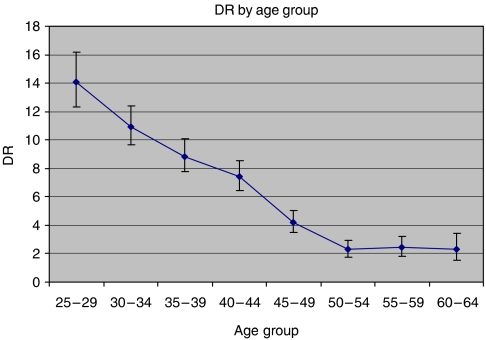
Detection rate (per 10^−4^ person-years) of CIN2+ by age class.

Cervical intraepithelial neoplasia 2+ DR (Table 2, Figure 1) was most frequent among 25- to 29-year-old women (DR=14.11, 95% CI: 12.30–16.18 per 10 000 person-years), and a statistically significant decreasing trend (Score test for trend of rates: *P*-value<0.0001) was observed with increasing age, the lowest DR being observed in the 50–54 (DR=2.28, 95% CI: 1.76–2.96) and in the 60–64 (DR=2.43, 95% CI: 1.85–3.19) age groups. Detection rate is rather stable (around 2 per 10 000 person-years) and significantly lower over 50 years of age.

When comparing 25–49 *vs* 50–64 age groups (Table 3), DR was constantly lower in the latter in all subgroups defined according to the number of previous negative screens. A small, statistically not significant, protective effect was observed in women above 50 years of age with more than one previous negative smear test (Score test for trend of rates: *P*-value>0.005).

Multivariable analysis when adjusting for screening programme, age <50 or ⩾50 years and interval between index and last negative smear demonstrate a statistically significant protective effect of four or more previous negative tests, but the effect of age ⩾50 years was much stronger (Table 4).

When comparing women <50 years of age with one previous negative test, the CR of CIN2+ (Table 5) is at least fivefold higher than in older women with one test, and raising to about eight if comparing younger women with one screen to women of age ⩾50 years with four tests.

When considering a cytological specificity of 0.97 and a histological specificity of 0.94 or the opposite combination, the cumulative probability of an FP CIN2+ is about 90, resulting as a sum of FP in each screening episode (Table 6). The ratio between TP and FP diagnosis is about 1 for women above 50 years of age who underwent one, two or three screening episodes, and it even reaches the value of 0.28 and 0.29 after four or five episodes, respectively (Table 6). However, in younger women this ratio is constantly above 10.44. The situation is similar when considering a cytological specificity of 0.96 and a histological specificity of 0.95 or its opposite combination. Actually, in this case, the cumulative probability of an FP CIN2+ is about 100; the ratio between TP and FP in women below 50 years of age ranges from 9.30 to 14.34 (i.e., it is lower than with the other combination) but in women above 50 years of age, the ratio is always below 1 and it reaches the value of 0.16 after four or more screening episodes.

## Discussion

In women ⩾50 years of age, the CR of CIN2+ is significantly lower compared to younger women, irrespective of the number of previous negative screening episodes. Combining the effect of age and the effect of the number of previous negative smears in the case of a CIN2+ diagnosis every 1 true-positive CIN2+ we may expect between 1 and 6.3 false-positive cases in the worst scenario, according to the combination in Table 6.

The present study is based on a large multicentric cohort and a great amount of person-years; nevertheless a number of points in the study design need to be discussed.

Ideally, the protective effect of cervical screening should be estimated on the risk of invasive carcinoma. Nevertheless, we chose CIN2+ as the outcome because in the whole cohort (287 330 women, 1110 CIN2+ cases), we found few invasive carcinomas (*n*=61) only. Incidence of cervical cancer in the study population is not comparable to that of the general population as women are selected for having performed at least two cytological screening tests, the first negative. Moreover, screening protocol addresses women with CIN2+ diagnosis to treatment.

Usually DR is calculated per smear as it is used to estimate a lesion prevalence, whereas here it was calculated as cases on person-years to take into account the screening history and the effect of previous tests on diagnosis.

We stratified the cohort choosing 50 years of age as cutoff. Even though the age groups have different lengths (25 and 15 years), this choice was based on previous studies showing a strong risk reduction for CIN2+ after 50 years of age (Gustafsson *et al*, 1995; Gram *et al*, 1998; International Agency for Research on Cancer, 2005). Also, our results show that DR of CIN2+ decreases significantly from 4.17 in the 45–49 age group to about 2 after 50 years of age (Table 2). When stratifying for 5-years age classes and previous screening episodes, after two, the DR before 50 years is doubled compared to older women (data not shown). To verify that the use of two groups of 25 and 15 years length would not affect our study conclusions, we estimated CR using 25–44 and 45–64 age groups, and the results show that the risk in younger women is at least threefold higher than that in older ones (the ratio ranging from 3.3 to 6.3).

As shown in Table 6, one critical point in cervical screening is the specificity of cytohistological diagnosis.

We estimated the joint probability of a false-positive CIN2+ diagnosis within a screening episode, as it depends on the probability of being a false-positive case at both the primary and the assessment test, for the adopted diagnostic category.

Several meta-analyses (Fahey *et al*, 1995; Nanda *et al*, 2000) have shown that cytological specificity ranges from 14 to 97% (Fahey *et al*, 1995). We assumed a specificity ranging from 0.94 to 0.97, according to estimates from studies on low-risk screening attenders (Nanda *et al*, 2000). Lower values did not allow the estimation of the true positives; anyway, it is very unlikely that a specificity as low as 0.60–0.70 might occur in a modern cytological screening setting (International Agency for Research on Cancer, 2005).

Histological diagnosis reproducibility might be as questionable as cytological diagnosis. Studies of cervical biopsies have shown fair-to-poor interobserver and intraobserver agreement in reporting (Robertson *et al*, 1989). We assumed histological specificity to be at least 0.94 according to the findings of ASCUS-LSIL Triage study (Stoler and Schiffman, 2001), which reviewed 2237 original histological slides.

According to these assumptions, the ratio between true-positive and false-positive results is almost above 10 under 50 years of age, whereas among older women, for each real case identified, one false positive is also diagnosed. After four tests, at least three false-positive cases are diagnosed every true positive (Table 6). These considerations hold also in the case of two 20-year age groups, that is 25–44 and 45–64 (data not shown). Yet, in our results, the effect is even stronger, thus demonstrating that the screening benefits over 50 years is uncertain.

False-positive results are associated with unnecessary assessment and its complications, adverse effects of treatment, unnecessary treatment, adverse effects of labelling or early diagnosis, anxiety and costs generated by investigations and treatment (International Agency for Research on Cancer, 2005). Hence an effort to increase specificity is needed, especially in older age groups.

A different CIN2+ DR is reported among the participating centres, likely due to real incidence differences and not due to diagnostic variability among laboratories. Actually, data on reproducibility that are available for cytological diagnosis in Italy showed that agreement was generally good (Montanari *et al*, 2003).

As the cohort includes women coming from six different Italian screening programmes, with different periods of observation, the CIN2+ DRs must be taken with caution. For this reason, we have performed an analysis on the Florence and Turin data only, as they contribute for 75% of the observed women. The estimates of the DRs, ratios and trends are very similar to those calculated on the whole cohort.

Centres with the highest CIN2+ incidence (Ravenna, Ferrara and Mantova) gave a limited contribution to the cohort as to person-years but they provided a great number of cases; this could have reduced the stronger effect observed in a previous analysis (Armaroli *et al*, 2005). Thus, decision makers should take into account the local CIN2+ prevalence when implementing local intervention strategies, as in single areas, the probability of being a case might be higher than that of being a false positive.

Women who undergo screening more frequently may have a lower risk of cervical disease because of a healthier lifestyle and a better access to treatment (Ronco *et al*, 1991). In the present study, DRs are low also because women were selected as having had at least one negative smear before the index test. Moreover, survival within the cohort is subordinated to not having shown a previous high-grade cervical lesion, as follow-up is stopped when a CIN2+ lesion occurs, that is the probability of being positive to the last Pap smear is conditional to having accumulated previous negative test.

Detection rates are affected by the duration of sojourn time and by the proportion of lesions that regress. Moreover, the sensitivity of smear test for long-sojourn time lesions as CIN2 (International Agency for Research on Cancer, 1986) depends on the number of smear tests performed during the sojourn time; on the other hand, frequent testing increases the DR of those significant lesions. This last effect may explain the observed risk reduction when the number of screening episodes increases, particularly after four negative tests.

The results of the present study are in agreement with other studies showing a risk reduction with increasing age (Cruickshank *et al*, 1997; Sasieni *et al*, 2003; Coldman *et al*, 2005) and the number of previous negative smear tests (Coldman *et al*, 2005).

Few cases of preinvasive lesions were diagnosed *ex novo* in well-screened women aged over 50 years (two or more 3- to 5-yearly negative screens) (Van Wijngaarden and Duncan, 1993) or in women with three consecutive (at most 3-yearly) negative screens before 50 years age (Cruickshank *et al*, 1997) or in women with at least three negative smear tests screens between 41 and 49 years of age (Gustafsson *et al*, 1995).

Considering invasive cervical cancer, a trend of decreasing risk with increasing number of smear tests is reported (International Agency for Research on Cancer, 1986; Gustafsson *et al*, 1995; Coldman *et al*, 2005).

In Turin (Italy), invasive carcinoma incidence during 1992–1998 was reduced by 75% in women who attended one screening at least, as compared to nonattenders (3.0 *vs* 9.5/100 000 person-years), the latter showing the highest incidence (Ronco *et al*, 2005).

The results of the present study, in accordance with other reports, may suggest that the adequacy of a routine screening test to identify early lesions in women over 50 years of age with at least four previous negative screens is questionable; possible alternative strategies may be explored. Women might be involved in the decision whether to stop screening or to undergo just another smear test in their life after evaluating the individual risk of a CIN2+ at further screening through algorithms based on age, screening history and living area-specific DRs. Comparing the future individual risk of being a case or a false positive may support and strengthen individual choices. The decision of stopping screening may also be supported by a negative result of HPV testing.

Informing women about the risk related to changes in their and their partners’ sexual habits (Brisson *et al*, 1994) may allow spontaneous return to the usual screening protocols.

Such strategies agree with the IARC recommendations for implementation on cervical cancer screening (International Agency for Research on Cancer, 2005) and with the IARC Working Group statement that there is little benefit from screening old women who have always tested negative in an organised screening programme. In particular, for women over 50 years of age, the Working Group recommend a 5-year screening interval.

The results of our study support the opinion that the benefit arising from cytological screening is uncertain in older women. Ethical and practical considerations subsequent to screening intensity reduction must be taken into account. A possible consequence might be an increase in invasive lesion incidence, compared to a major resource saving. Estimates of unprevented cervical cancers are in the magnitude of about two cases per 100 000 person-years (Sherlaw-Johnson *et al*, 1999). It is thus desirable to evaluate if benefits arising from saved resource allocation to more cost-effective interventions would make acceptable to reduce or to stop screening in 50-year-old or older women with a negative documented screening history.

## Figures and Tables

**Table 1:** Distribution of the study population and DR (with 95% CI) by screening programme

**Screening programme**	**Starting year**	**No. of women (%)**	**Person-years**	**No. of cases**	**DR (95% CI)**
Firenze	1986	162 314 (56)	1 273 442	824	6.47 (6.04–6.93)
Torino	1992	54 367 (19)	225 546	69	3.06 (2.42–3.87)
Mantova	1993	23 571 (8)	92 632	79	8.53 (6.84–10.63)
Ravenna	1995	19 476 (7)	55 933	57	10.19 (7.86–13.21)
Ferrara	1996	22 181 (8)	52 011	80	15.38 (12.36–19.15)
Viterbo	1997	5421 (2)	14 986	1	0.67 (0.09–4.74)
Total	—	287 330	1 714 550	1110	

CI=confidence interval; DR=detection rate.

**Table 2:** Detection rate (per 10^−4^ person-years) of CIN2+ by age class

**Age class (years)**	**No. of cases (*N*=1110)**	**Person-years (*N*=1 714 550)**	**DR (95% CI) (per 10^−4^ person-years)**
25–29	205	145 296	14.11 (12.30–16.18)
30–34	252	229 902	10.96 (9.69–12.40)
35–39	217	245 243	8.85 (7.75–10.11)
40–44	192	259 391	7.40 (6.43–8.53)
45–49	111	265 922	4.17 (3.47–5.03)
50–54	57	249 725	2.28 (1.76–2.96)
55–59	52	213 905	2.43 (1.85–3.19)
60–64	24	105 166	2.28 (1.53–3.41)

CI=confidence interval; CIN=cervical intraepithelial neoplasia; DR=detection rate.

Score test for trend of rates: *P*-value<0.0001.

**Table 3:** Detection rate (per 10^−4^ person-years) of CIN2+ by number of previous screening episodes

**Age class (years)**	**No. of previous screening episodes**	**No. of cases (*N*=1110)**	**Person-years (*N*=1 714 550)**	**DR (95% CI) (per 10^−4^ person-years)**	**DR ratio (95% CI)**
All ages	1	594	933 165	5.98 (5.49–6.47)	1
	2	300	420 778	7.19 (6.37–8.01)	1.12 (0.98–1.29)
	3	131	197 909	7.58 (6.04–9.12)	1.04 (0.86–1.26)
	4	46	92 676	7.43 (3.29–11.58)	0.78 (0.58–1.05)
	5+	39	70 022	6.32 (4.04–8.60)	0.87 (0.63–1.21)
					
25–49	1	518	634 520	8.16 (7.49–8.90)	1
	2	268	280 039	9.57 (8.49–10.79)	1.17 (1.01–1.36)
	3	114	129 361	8.81 (7.34–10.59)	1.08 (0.88–1.32)
	4	41	58 929	6.96 (5.12–9.45)	0.85 (0.62–1.17)
	5+	36	42 905	8.39 (6.05–11.63)	1.03 (0.73–1.44)
					
50–64 s	1	76	298 645	2.55 (2.03–3.19)	1
	2	32	140 738	2.27 (1.61–3.22)	0.89 (0.59–1.35)
	3	17	68 548	2.48 (1.54–3.99)	0.98 (0.58–1.65)
	4	5	33 747	1.48 (0.62–3.56)	0.58 (0.24–1.44)
	5+	3	27 117	1.11 (0.36–3.43)	0.44 (0.14–1.38)

CI=confidence interval; CIN=cervical intraepithelial neoplasia; DR=detection rate.

Standardised and stratified by age (25–49 years, 50–64 years).

Score test for trend of rates: *P*-value>0.5.

**Table 4:** Relative risk (hazard ratios) of CIN2+ and 95% CI, adjusted for screening programme and interval between index and last screening episode

**Factors**	**Level**	**Relative risk (95% CI)**
No. of previous screening episodes	1 (referent)	1
	2	1.10 (0.95–1.28)
	3	0.96 (0.78–1.19)
	4	0.70 (0.51–0.97)
	5	0.65 (0.45–0.92)
Age	25–49 years (referent)	1
	50–64 years	0.29 (0.24–0.35)

CI=confidence interval; CIN=cervical intraepithelial neoplasia.

Test for trend on risks referring to *n* screening episodes: *P*-value>0.5.

**Table 5:** Cumulative risk (per 100) between 25 and 49 years and 50 and 64 years of CIN2+

	**Cumulative risk (per 100) (95% CI)**	
**No. of previous negative screening episodes**	**25–49 years**	**50–64 years**
1	2.06 (1.88–2.23)	0.36 (0.28–0.45)
2	2.46 (2.15–2.78)	0.38 (0.24–0.52)
3	2.74 (2.01–3.47)	0.36 (0.17–0.54)
4	3.05 (0.74–5.30)	0.23 (0.00–0.46)
5+	2.06 (1.26–2.85)	0.23 (0.00–0.52)

CI=confidence interval; CIN=cervical intraepithelial neoplasia.

**Table 6:** Cumulative risk (CR), probabilities of cytological and histological false-positive diagnosis and of true positive per 10 000, ratio between true-positive and false-positive, ratio between CR and false-positive, by screening episodes, for different combination of cytological and histological specificities

	**CR (per 10 000)**			**True positive (per 10 000)**		**True positive/false positive**		**Positive/false positive**	
**No. of screening episodes**	**25–49 (years)**	**50–64 (years)**	**False positive (per 10 000)**	**25–49 (years)**	**50–64 (years)**	**25–49 (years)**	**50–64 (years)**	**25–49 (years)**	**50–64 (years)**
*Cytological specificity: 0.97, histological specificity: 0.94 or cytological specificity: 0.94, histological specificity: 0.97*									
1	206	36	18.00	188.00	18.00	10.44	1	11.44	2
2	246	38	17.97	228.03	20.03	12.69	1.11	13.69	2.11
3	274	36	17.94	256.06	18.06	14.28	1.01	15.28	2.01
4	305	23	17.90	287.10	5.10	16.04	0.28	17.04	1.28
5	206	23	17.87	188.13	5.13	10.53	0.29	11.53	1.29
									
*Cytological specificity: 0.96, histological specificity: 0.95 or cytological specificity: 0.95, histological specificity: 0.96*									
1	206	36	20.00	186.00	16.00	9.30	0.80	10.30	1.80
2	246	38	19.96	226.04	18.04	11.32	0.90	12.32	1.90
3	274	36	19.92	254.08	16.08	12.75	0.81	13.75	1.81
4	305	23	19.88	285.12	3.12	14.34	0.16	15.34	1.16
5	206	23	19.84	186.16	3.16	9.38	0.16	10.38	1.16
